# Acute Vaginal Bleeding as the Initial Manifestation of Multifactorial Thrombocytopenia: A Case Report

**DOI:** 10.7759/cureus.106820

**Published:** 2026-04-10

**Authors:** Alireza Izadian Bidgoli, Shabnam Yazdanpanah, Jordan De Guzman, Atharv Joshi, Alberto Gomez Veliz

**Affiliations:** 1 Internal Medicine, American University of the Caribbean School of Medicine, Cupecoy, SXM; 2 Internal Medicine, Jackson Memorial Hospital, Miami, USA

**Keywords:** abnormal uterine bleeding, drug-induced thrombocytopenia, multifactorial hemostasis, platelet dysfunction, valproate

## Abstract

Thrombocytopenia is a common hematologic abnormality with a broad differential diagnosis, often requiring careful evaluation to identify overlapping and potentially reversible etiologies. Medication-induced thrombocytopenia is well recognized, particularly with valproic acid, while substance use such as cocaine may further exacerbate bleeding through platelet dysfunction and vascular effects. We present the case of a 39-year-old woman with a history of seizure disorder treated with valproic acid and comorbid psychiatric illness, including a prior suicide attempt, who presented with acute heavy vaginal bleeding. Laboratory evaluation revealed thrombocytopenia without evidence of hemolysis or coagulopathy. Peripheral smear was unremarkable aside from nonspecific red blood cell changes, and imaging studies did not demonstrate hepatosplenomegaly or structural abnormalities. Further history revealed recent cocaine use, raising concern for a multifactorial process. Valproic acid was discontinued due to suspected drug-induced thrombocytopenia, and the patient was transitioned to an alternative antiepileptic regimen. With supportive care and cessation of the offending agents, the patient demonstrated stabilization of platelet counts and resolution of bleeding. This case highlights the importance of recognizing multifactorial thrombocytopenia, particularly in patients with concurrent medication exposure and substance use. Early identification of reversible contributors is critical, as prompt discontinuation of offending agents can lead to rapid clinical improvement and prevent unnecessary invasive evaluation. Additionally, this case underscores the need for a multidisciplinary approach that integrates hematologic, neurologic, and psychiatric considerations in complex presentations while comparing the case with the current literature.

## Introduction

Thrombocytopenia is a hematologic condition characterized by decreased platelet production or increased peripheral destruction, resulting in impaired primary hemostasis and an increased risk of bleeding [1,2]. Platelets play a central role in primary hemostasis by forming plugs at sites of vascular injury, and disruptions in platelet number or function can lead to clinically significant bleeding [2]. While thrombocytopenia may be asymptomatic in mild cases, more severe reductions in platelet count can result in life-threatening bleeding, particularly when additional hemostatic abnormalities are present [3]. Thrombocytopenia is defined as a platelet count below 150,000 per microliter, with normal values ranging from 150,000 to 450,000 [2,4].

Medication-induced thrombocytopenia is a well-recognized and clinically significant etiology, accounting for a substantial proportion of acquired cases [1,5]. Valproic acid, a commonly used antiepileptic agent, has been associated with both dose-dependent bone marrow suppression and qualitative platelet dysfunction, leading to impaired platelet production and aggregation [5]. In addition to quantitative platelet abnormalities, qualitative platelet dysfunction represents an important and often underrecognized contributor to bleeding risk [1,2]. Cocaine use has been associated with endothelial injury, vasoconstriction, and platelet dysfunction, further compromising primary hemostasis and increasing bleeding risk [1]. When these factors coexist, they may result in clinically significant bleeding that exceeds expectations based on platelet count alone [1,3].

We report a case of acute heavy vaginal bleeding in a patient with multifactorial thrombocytopenia, in which valproic acid-induced platelet suppression was compounded by cocaine-associated platelet dysfunction. This case highlights the importance of recognizing interacting mechanisms of impaired hemostasis and underscores the need to consider multifactorial etiologies when clinical severity is not fully explained by laboratory findings.

## Case presentation

Clinical presentation

A 39-year-old woman with a past medical history significant for asthma, bipolar/schizophrenia spectrum disorder, prior suicide attempt, and generalized tonic-clonic seizure disorder presented with a four-day history of progressively worsening heavy vaginal bleeding. She reported passage of multiple large clots, significantly increased from her baseline menstrual pattern, accompanied by fatigue and generalized weakness. She denied prior episodes of similar severity.

Her history was notable for ongoing treatment with valproic acid (divalproex) for seizure control and intermittent use of cocaine and marijuana. On further evaluation, the patient endorsed recent psychosocial stressors and expressed passive suicidal ideation without an active plan at the time of presentation.

Evaluation (history, examination, laboratory findings, and imaging)

On presentation, the patient was hemodynamically stable and in no acute distress. Physical examination revealed a well-appearing female with moist mucous membranes and no scleral icterus. Cardiopulmonary examination was unremarkable. Abdominal examination demonstrated a soft, non-tender, non-distended abdomen without palpable organomegaly. No peripheral edema was noted. Cutaneous examination revealed no petechiae, purpura, or other overt bleeding manifestations. Neurological examination was non-focal.

Initial laboratory evaluation demonstrated thrombocytopenia with a platelet count of 93 ×10³/µL, hemoglobin of 12.6 g/dL, and a white blood cell count of 11.1 ×10³/µL. Coagulation studies were within normal limits, including an INR of 0.96, arguing against an underlying coagulopathy. A hemolysis workup revealed an LDH of 251 U/L, haptoglobin of 208 mg/dL, and total bilirubin of 0.4 mg/dL, with no laboratory evidence of hemolysis. Peripheral blood smear demonstrated no schistocytes or platelet clumping, reducing the likelihood of thrombotic microangiopathy or pseudothrombocytopenia.

Serial laboratory monitoring revealed a progressive decline in platelet count from 93 ×10³/µL on admission to a nadir of 39 ×10³/µL during hospitalization, without a corresponding drop in hemoglobin or evidence of hemolysis. Renal function remained preserved throughout hospitalization, further arguing against systemic processes such as thrombotic microangiopathy. A comprehensive summary of laboratory findings, including serial platelet trends, is provided in Table 1.

**Table 1:** Summary of laboratory findings at presentation and during hospitalization. Values are presented as measured at admission and as peak or range values observed during hospitalization. Reference ranges are provided for comparison. Platelet counts demonstrated a progressive decline during hospitalization, supporting a dynamic thrombocytopenic process. WBC: white blood cell; MCV: mean corpuscular volume; BUN: blood urea nitrogen; AST: aspartate aminotransferase; ALT: alanine aminotransferase; LDH: lactate dehydrogenase; INR: international normalized ratio; PT: prothrombin time; aPTT: activated partial thromboplastin time.

Laboratory Test	At Admission	During Hospitalization (Peak/Range)	Reference Range (Unit)
Hemoglobin	12.6	13.2–13.4	13.5–17.5 g/dL
White Blood Cell Count	11.1	5.5–9.2	4.0–11.0 ×10³/µL
Platelet Count	93	39–68	150–400 ×10³/µL
Mean Corpuscular Volume (MCV)	97.5	95.1–98.3	80–100 fL
Creatinine	0.57	0.57–0.70	0.6–1.3 mg/dL
Blood Urea Nitrogen (BUN)	15	13–18	7–20 mg/dL
Sodium	134	133–136	135–145 mmol/L
Potassium	4.1	3.6–4.4	3.5–5.0 mmol/L
Calcium	8.3	8.3–8.8	8.5–10.5 mg/dL
Total Protein	6.2	5.8–6.2	6.0–8.3 g/dL
Albumin	3.3	3.1–3.3	3.5–5.0 g/dL
Total Bilirubin	0.4	0.4–0.8	0.2–1.2 mg/dL
AST (SGOT)	25	32–36	10–40 U/L
ALT (SGPT)	12	14–16	7–56 U/L
LDH	251	251–341	140–280 U/L
Haptoglobin	208	—	30–200 mg/dL
INR	0.96	0.85–0.96	0.8–1.2
PT (Prothrombin Time)	13	11.9–13.0	11–13.5 sec
aPTT	26	26–29	25–35 sec

Additional evaluation included abdominal ultrasound, which demonstrated normal hepatic echotexture and a spleen measuring approximately 10 cm, without evidence of splenomegaly or portal hypertension. No intra-abdominal pathology was identified to account for the patient’s thrombocytopenia or bleeding.

Treatment and diagnosis

Hematology was consulted for evaluation of thrombocytopenia in the setting of active vaginal bleeding. The absence of hemolysis, normal coagulation studies, and lack of morphologic abnormalities on peripheral smear made thrombotic microangiopathy and coagulopathic processes unlikely. The normal INR further localized the bleeding diathesis to a platelet-mediated process.

**Figure 1:**
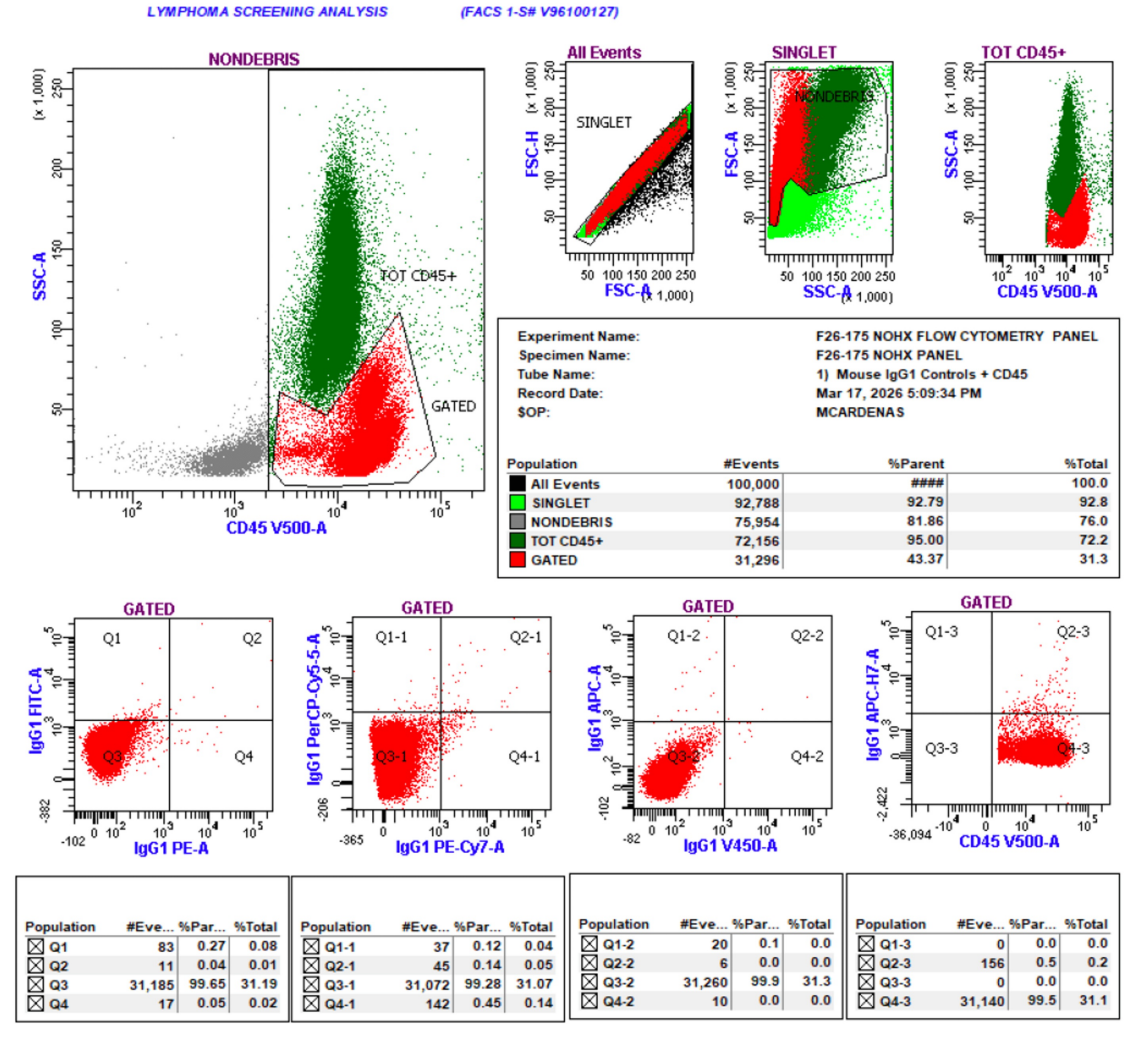
Peripheral blood flow cytometry. Flow cytometry demonstrating no abnormal blast population and no immunophenotypic evidence of a clonal lymphoid or leukemic process.

Flow cytometry (Figure 1) demonstrated no abnormal blast population and no immunophenotypic evidence of a clonal lymphoid or leukemic process, effectively excluding an underlying hematologic malignancy.

Given the progressive decline in platelet count and the absence of alternative etiologies, a multifactorial process was suspected. The patient had been receiving valproic acid for seizure management, a medication known to cause dose-dependent thrombocytopenia and platelet dysfunction. Additionally, recent cocaine use raised concern for concurrent platelet dysfunction and endothelial injury, further impairing primary hemostasis.

Valproic acid was discontinued, and the patient was transitioned to an alternative antiepileptic regimen. Following cessation of the offending agent and supportive management, platelet counts stabilized and clinical bleeding improved, supporting a reversible drug-induced etiology with contributing multifactorial mechanisms.

Outcome

During hospitalization, the patient demonstrated progressive clinical improvement, with stabilization of platelet count and resolution of active vaginal bleeding following discontinuation of valproic acid and supportive management. She remained hemodynamically stable throughout her course.

Her psychiatric status was reassessed prior to discharge, and she denied active suicidal intent, with a safety plan established. The patient was discharged in stable condition with outpatient follow-up arranged with hematology, neurology, and psychiatry. Peripheral blood flow cytometry was obtained to exclude an occult hematologic malignancy and was unrevealing, with no abnormal blast population or clonal lymphoid population identified. This further supported a non-neoplastic explanation for the thrombocytopenia and reduced concern for marrow-infiltrative or leukemic processes.

## Discussion

Background 

Abnormal uterine bleeding (AUB) is a common gynecologic condition in women of reproductive age, defined by bleeding of abnormal volume, duration, or frequency. AUB can be broadly categorized into structural and non-structural etiologies. Among the non-structural etiologies are thrombocytopenia and platelet dysfunction, with drug-induced thrombocytopenia being a well-established mechanism for AUB. One example, valproic acid, a commonly used antiepileptic medication, is associated with bone marrow suppression leading to thrombocytopenia. A critical review of available data reported prevalence ranges from 5% to 54%, with rates of approximately 12% to 18% in larger studies. Furthermore, the effect appears more frequently observed in certain populations, including females and those receiving higher dose concentrations [6]. Despite its effects on platelet production and function, clinically significant bleeding is relatively uncommon with mild to moderate thrombocytopenia (platelet count >50,000/μL), suggesting that additional factors should be considered when bleeding appears disproportionate to the degree of platelet reduction [7].

In addition, cocaine use has been associated with alterations in hemostasis, including vasoconstriction, endothelial injury, and platelet dysfunction [8]. While these effects are recognized, their combined impact with valproic acid-induced thrombocytopenia has not been well characterized. The interaction of these factors may contribute to AUB and should be considered when bleeding appears disproportionate to laboratory findings.

Although both valproic acid-induced thrombocytopenia and cocaine-associated platelet dysfunction are individually well described, their combined impact on hemostasis and contribution to clinically significant AUB remain poorly characterized.

We report this case to highlight the potential for synergistic impairment of hemostasis, specifically in the setting of valproic acid-induced thrombocytopenia and cocaine-associated platelet dysfunction. This combination may present as clinically significant AUB despite only mild thrombocytopenia.

Physiology/pathophysiology 

It is known that valproic acid can suppress platelet production by affecting megakaryocytes in the bone marrow, as well as circulating platelets, impairing primary hemostasis both qualitatively and quantitatively [9,10]. Valproic acid-associated thrombocytopenia is dose and concentration-dependent; therefore, higher titer levels are predictive of a greater risk of thrombocytopenia [11]. Studies have shown that this is mediated through altered hematopoietic differentiation, leading to lower megakaryocyte production [9]. Additionally, it’s been found that valproate induces platelet dysfunction by reducing fibrinogen binding to glycoprotein IIb/IIIa receptors and increasing expression of CD62P, reducing platelet aggregation [9,10]. This combination effect explains why some patients exceed the expectations of bleeding severity compared to what is normally seen when looking at low platelet count alone. Currently, there are no known genetic mutations or proliferative pathway mechanisms involved in valproate-induced thrombocytopenia, and this condition is likely mediated through reversible pharmacological effects [9,10].

The patient’s presentation with heavy vaginal bleeding and passage of clots is consistent with impaired primary hemostasis. Both the reduced platelet count (93 ×10³/µL) and qualitative platelet dysfunction contributed to ineffective hemostatic plug formation [9,10]. Given the cyclical endometrial shedding and high vascular turnover of the uterus, disruptions in primary hemostasis can result in clinically significant bleeding [12]. Notably, the patient’s normal INR supports the absence of a coagulation factor deficiency and further localizes the bleeding diathesis to a platelet-mediated process.

Laboratory findings further showed no anemia or leukopenia, with only isolated thrombocytopenia supporting impaired production due to drug side effects rather than bone marrow infiltration. Normal coagulation studies and the absence of schistocytes excluded a microangiopathic process, and peripheral smear findings ruled out pseudothrombocytopenia. Unlike immune thrombocytopenia (ITP), valproate-associated thrombocytopenia is primarily mediated by dose-dependent bone marrow suppression and qualitative platelet dysfunction [9,10]. The absence of large or giant platelets on peripheral smear further argues against ITP. Additionally, the absence of petechiae, purpura, or other diffuse mucocutaneous bleeding manifestations further decreased the likelihood of ITP. Most notably, the patient’s clinical improvement following discontinuation of valproic acid supports a reversible drug-induced mechanism rather than a primary hematologic disorder.

Comparative analysis of our case with the current literature

Clinical Presentation

Heavy vaginal bleeding and passage of large clots are not a common presentation of valproate-induced thrombocytopenia. Previous studies have shown that this drug-induced thrombocytopenia commonly presents as incidental platelet decline or mild bleeding, with increased risk observed in women and at higher drug exposure levels [11]. Abnormal vaginal bleeding with valproate is usually endocrine-mediated; however, heavy vaginal bleeding may also occur due to drug-induced thrombocytopenia and platelet dysfunction and can present similarly to other hematologic disorders, making it clinically significant [12]. This presentation could be mistaken for ITP or other microangiopathic processes. Normal coagulation studies, absence of schistocytes on peripheral smear, and the temporal relationship to validate use helped exclude these conditions [10-12].

*Diagnostic Workup* 

Valproate-induced thrombocytopenia was a diagnosis of exclusion in this patient. Normal coagulation studies, absence of schistocytes on peripheral smear, and preserved renal function reduced the likelihood of thrombotic microangiopathies [9-13]. Additionally, gynecologic causes of abnormal uterine bleeding, including pregnancy and structural pathology, were considered and excluded based on clinical evaluation and imaging. In this context, the temporal association with valproic acid use and subsequent clinical improvement following its discontinuation supported a reversible drug-induced etiology [10,11].

Peripheral smear findings in this case demonstrated no schistocytes or platelet clumping, further reducing the likelihood of thrombotic thrombocytopenic purpura (TTP) and pseudothrombocytopenia. The absence of hemolysis preserved renal function and lack of neurologic deficits further argue against TTP [13]. Additionally, abdominal imaging revealed no splenomegaly, making splenic sequestration unlikely.

Flow cytometry (Figure 1) was performed, which returned negative. The primary diagnostic challenge remained distinguishing drug-induced thrombocytopenia with platelet dysfunction from ITP, which remains a diagnosis of exclusion based on clinical and laboratory findings [14,15]. Given the patient’s improvement after stopping valproate, the laboratory profile was more consistent with a non-immune, reversible drug-related process.

*Management* 

Standard management of valproate-associated thrombocytopenia involves dose reduction or discontinuation of the offending agent and close monitoring [10,11]. This approach is supported by studies demonstrating improvement in platelet counts following discontinuation of valproic acid [10,11]. In this case, management aligned with recommended approaches for drug-induced thrombocytopenia. Valproic acid was discontinued, and the patient was transitioned to an alternative antiepileptic agent, with close monitoring of serial platelet counts. The decision to withhold platelet transfusion and immunomodulatory therapy was appropriate given the patient’s hemodynamic stability, absence of severe thrombocytopenia, and lack of evidence supporting an immune-mediated or thrombotic microangiopathic process.

*Outcome* 

Resolution of vaginal bleeding and stabilization of platelet count following discontinuation of valproic acid is consistent with the expected reversible nature of drug-induced thrombocytopenia [11]. In contrast, ITP often follows a relapsing or chronic course requiring ongoing management, and TTP carries significant morbidity and mortality despite improved outcomes with early intervention [13,14]. The patient was discharged in stable condition with outpatient follow-up arranged with hematology, neurology, and psychiatry for continued monitoring.

What we learned from this case 

This case highlights that the severity of bleeding may not correlate directly with platelet count and underscores the importance of recognizing qualitative platelet dysfunction in clinical assessment. Although the degree of thrombocytopenia was moderate, the patient’s presentation suggests that additional factors affecting platelet function may significantly influence bleeding risk.

Determining the underlying cause of thrombocytopenia can be particularly challenging, as the differential diagnosis is broad and often includes overlapping etiologies such as immune-mediated processes, medication effects, and systemic disorders. Failure to accurately identify the primary contributor may lead to misdiagnosis and inappropriate management. In this case, careful integration of clinical history, laboratory data, and medication exposure was essential in distinguishing a reversible drug-induced process from other potential causes.

These findings emphasize the need to consider multifactorial etiologies in patients with thrombocytopenia, particularly in the presence of medication exposure and substance use. A focused and systematic evaluation is critical, as early identification and removal of reversible contributors may result in rapid clinical improvement without the need for invasive diagnostic procedures or immunosuppressive therapy.

Overall, this case reinforces that effective management of thrombocytopenia-related bleeding requires an integrated approach that accounts for both quantitative and qualitative abnormalities in hemostasis, especially when clinical presentation appears discordant with laboratory findings.

## Conclusions

This case illustrates that clinically significant bleeding may occur in the setting of moderate thrombocytopenia when both quantitative and qualitative platelet abnormalities are present. The patient’s presentation was not fully explained by platelet count alone, but rather likely reflects a multifactorial process involving valproic acid-associated platelet suppression and cocaine-related platelet dysfunction. The observed clinical improvement following discontinuation of valproic acid supports a reversible drug-induced component, while the contribution of concurrent substance use may have further impaired hemostasis. This case underscores the importance of considering multifactorial etiologies in patients with thrombocytopenia, particularly when clinical severity appears discordant with laboratory findings. Recognition of potential interacting mechanisms may help guide timely diagnosis and management, although further study is needed to better characterize these relationships.
